# *Elatostema weizhianum*, a new species of Urticaceae from Zhejiang, East China

**DOI:** 10.3897/phytokeys.277.200946

**Published:** 2026-07-07

**Authors:** Yue-Liang Xu, Zi-Hong Zheng, Yi-Fei Lu, Zhan-Sheng Tang, Xiao-Feng Jin

**Affiliations:** 1 Zhejiang Museum of Natural History, Hangzhou, Zhejiang, 310012, China Zhejiang Museum of Natural History Hangzhou China; 2 Administration of Zhejiang Jiulongshan National Natural Reserve, Suichang, Zhejiang, 323300, China Administration of Zhejiang Jiulongshan National Natural Reserve Suichang China; 3 School of Forestry and Bio-technology, Zhejiang A&F University, Hangzhou, Zhejiang, 311300, China School of Forestry and Bio-technology, Zhejiang A&F University Hangzhou China

**Keywords:** *
Elatostema
*, endemic species, infrageneric division, limestone zone, taxonomy

## Abstract

The genus *Elatostema* (Urticaceae) is most diverse in the limestone regions of southern China. *Elatostema weizhianum*, a new species from Zhejiang Province, East China, is described and illustrated in the present paper. The phylogenetic analysis revealed that the new species is closely related to *E. hypoglaucum* but differs in having a triplinerved leaf base, stipules 4–6 mm long and purple-brown maculate, peduncles of staminate inflorescences 5–12 mm long and densely strigose, and achenes ca. 1 mm long, longitudinally 5-ribbed, and tuberculate. This new species is morphologically similar to *E. grandidentatum* but differs in having purple-brown maculate stipules, subsessile pistillate inflorescences borne in pairs axillary, a glabrous receptacle, triangular bracts that are sparsely pubescent dorsally, ovate-orbicular outer bracteoles, oblong-navicular inner bracteoles that are corniculate at the apex and pubescent dorsally and at the margin, inconspicuous, degenerative sepals, and 5-ribbed and tuberculate achenes. Based on current observations of the geographic range and population size of the new species, it was assessed as “EN” under the IUCN categories and criteria.

## Introduction

The Urticaceae, comprising more than 2,500 species across 59 accepted genera, is traditionally divided into five tribes (Friis 1993; Wang and Chen 1995; Chen et al. 2003, 2020; Conn and Hadiah 2009; POWO 2025). In China, the family is represented by 26 genera and 400 species (Chen et al. 2003, 2020; Deng et al. 2013; Fu et al. 2026).

The genus *Elatostema*, belonging to the tribe Elatostemateae Gaudich. (Wang and Chen 1995; Wang 2010), consists of 659 species primarily distributed in tropical and subtropical regions of Asia, Africa, and Oceania (Lin et al. 2003; Wang 2014; POWO 2025). Its diversity centers are concentrated in two regions: (1) karst areas of southern China (184 species, particularly in southeastern Yunnan, western and northern Guangxi, and southern Guizhou), and (2) Southeast Asian archipelagos, including the Philippines (ca. 90 species), New Guinea (ca. 67 species), Borneo (ca. 42 species), Java (ca. 15 species), and Sulawesi (ca. eight species) (Wang 2014).

In China, *Elatostema* exhibits exceptionally high endemism (Wang and Chen 1995; Wang 2014). Among its 280 endemic species, most are narrowly distributed and restricted to single mountainous regions or counties (Wang 2014; Fu et al. 2019, 2021). Yunnan and Guangxi are the provinces with the highest species richness, hosting 151 species (106 endemic) and 99 species (65 endemic), respectively (Wang 2014).

A recent study on the phylogeny of *Elatostema* sensu lato (s.l.; including *Elatostema* sensu stricto [s.s.] and related genera; Tseng et al. 2019) revealed three well-supported clades, which were recognized as *Procris*, *Elatostematoides*, and *Elatostema* sensu auct. (s.a.). Within *Elatostema* s.a., molecular phylogeny showed four strongly supported clades, and the core *Elatostema* contained most species of the morphologically defined *Elatostema* (Tseng et al. 2019). Because of the large number of species but limited collections, *Elatostema* is recognized as a taxonomically difficult group. Different opinions on the morphological delimitation of its species have resulted in taxonomic instability. Integrative taxonomy with field collection, morphological observation, and phylogenetic analysis of some Chinese limestone karst *Elatostema* species appears to be an effective and accessible approach (Xin et al. 2023).

During a field survey in Zhejiang Province, eastern China, a distinct *Elatostema* species was discovered and is described as a new species below.

## Material and methods

### Taxon sampling, DNA extraction, and sequencing

A total of 103 species were included in this study for phylogenetic analyses, comprising 92 species representing *Elatostema*. Of these, only the new species was newly sequenced; the remaining sequences (99 ITS, 100 *psbA-trnH*, and 101 *psbM-trnD*) were sourced from the NCBI GenBank database (accession numbers listed in Appendix 1). Genomic DNA was extracted from silica gel-dried leaves using the CTAB method. Paired-end libraries were prepared and sequenced on the DNBSEQ-T7 platform (China National GenBank, Shenzhen, China), yielding 150 bp paired-end reads. Three retrieved datasets were used as references to extract ITS, *psbA-trnH*, and *psbM-trnD* from the genome skimming data of the new species using GeneMiner2 (Xie et al. 2024). Newly generated data were deposited in the China National Center for Bioinformation-National Genomics Data Center (CNCB-NGDC) under BioProject accession no. PRJCA035390 and BioSample accession nos. SAMC4600365 (*E. weizhianum* one, staminate individual, voucher specimen: *Y.L. Xu & F.G. Zhang 1493*) and SAMC4600366 (*E. weizhianum* one, pistillate individual, voucher specimen: *Y.L. Xu & al. 1572*).

### Morphological observations

Specimens of the new species were collected from Suichang and Longquan counties in southwestern Zhejiang Province, East China. Subsequently, the relevant literature was carefully studied, and the type specimens of the related species *E. grandidentatum* W.T.Wang (Wang and Chen 1979), *E. longibracteatum* W.T.Wang (Wang 1982), and *E. albovillosum* W.T.Wang (Wang 2010) were examined in both PE and IBK. The following characters—indumentum on stems, leaves, and receptacles; venation and serration of leaves; shapes of bracts and bracteoles of pistillate inflorescences; and achene surface—were critically compared.

### Phylogenetic analyses

Sequence alignments for ITS, *psbA-trnH*, and *psbM-trnD* were performed using the MAFFT online server (https://mafft.cbrc.jp/alignment/server/) (Katoh et al. 2019), followed by manual trimming in MEGA v.7 (Kumar et al. 2016). The optimal nucleotide substitution model for each dataset was identified using ModelFinder v.2.2.0 (Kalyaanamoorthy et al. 2017). Concatenation of aligned sequences was executed in PhyloSuite (Zhang et al. 2020). Phylogenetic relationships were inferred through both maximum likelihood (ML) and Bayesian inference (BI) methods, with *Boehmeria densiflora*, *Nanocnide japonica*, and *Poikilospermum acuminatum* designated as outgroups. The ML analysis was implemented in RAxML-HPC BlackBox v.8.2.12 (Stamatakis 2014) via the CIPRES Science Gateway v.3.3, employing the GTR+G model and 1,000 bootstrap replicates. The BI analysis was conducted in MrBayes v.3.2.7a (Ronquist et al. 2012) using two independent Markov chain Monte Carlo (MCMC) runs, each comprising four chains iterated for 50 million generations, with trees sampled every 1,000 generations. Convergence was assessed using the average standard deviation of split frequencies (< 0.01), and the first 25% of trees were discarded as burn-in prior to generating a 50% majority-rule consensus tree.

## Results

### Phylogenetic reconstruction

**Figure 1.**
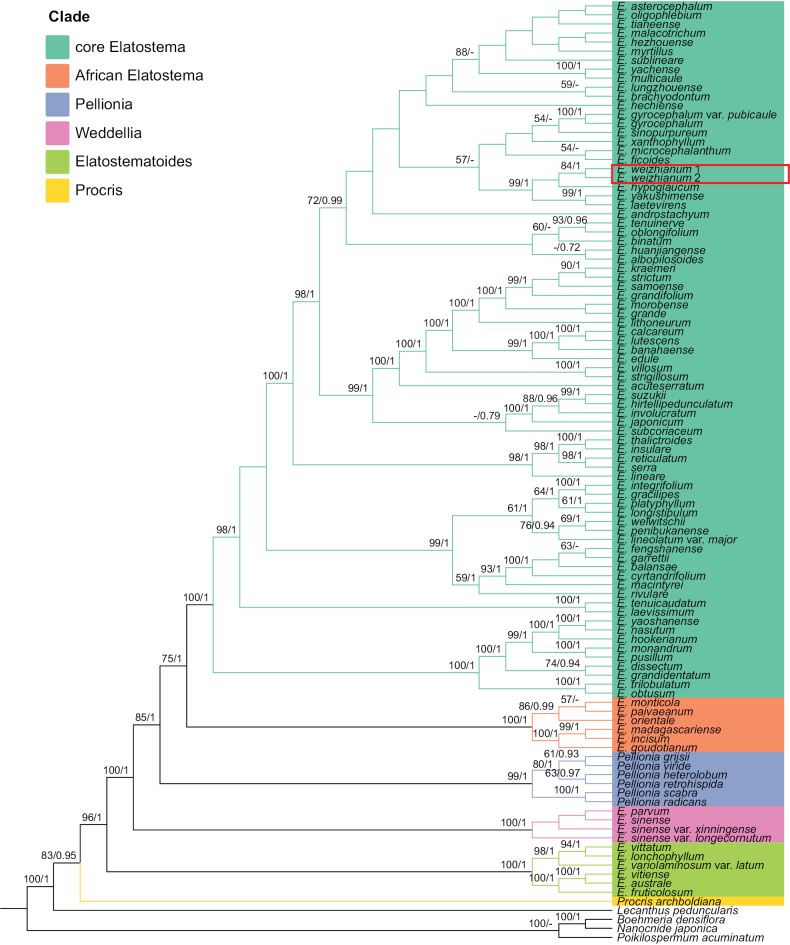
Phylogenetic relationships of *Elatostema* inferred from ITS, *psbA-trnH*, and *psbM-trnD* based on ML and BI methods. Bootstrap values (BS) and posterior probability values (PP) are shown above the branches; only BS > 50% or PP > 0.7 are shown.

The aligned lengths of ITS, *psbA-trnH*, and *psbM-trnD* were 1,152 bp, 632 bp, and 763 bp, respectively. Based on the lowest Akaike information criterion (AIC) scores, the GTR+F+G4 model was selected for both *psbA-trnH* and *psbM-trnD*, while the SYM+I+G4 model was chosen for ITS. Phylogenetic trees inferred by ML and BI methods exhibited largely congruent topologies. Consequently, only the ML tree is presented (Fig. 1). Within *Elatostema* s.l., six major clades were identified: the *Procris* clade, the *Elatostematoides* clade, the *Weddellia* clade, the *Pellionia* clade, the African *Elatostema* clade, and the core *Elatostema* clade. The two accessions of the new species formed a strongly supported clade (BS = 84%; PP = 1) within the core *Elatostema* clade. Phylogenetic analysis revealed that the new species (two samples) is most closely related to *E. hypoglaucum*, while exhibiting a distant relationship to *E. grandidentatum*—its morphologically most similar species. The molecular phylogenetic evidence strongly supports the recognition of *E. weizhianum* as a distinct species, and the morphological similarity reveals the infrageneric position of this species.

### Taxonomic treatment

#### Elatostema weizhianum

Taxon classificationPlantaeRosalesUrticaceae

Y.L.Xu & X.F.Jin sp. nov.

B52EB28E-5537-5473-988E-9281914E2733

urn:lsid:ipni.org:names:77382774-1

Figs 2, 3

[Elatostema sect. *Elatostema* ser. *Papillosa* W.T.Wang]

##### Diagnosis.

This new species is closely related to *E. hypoglaucum* B.L.Shih & Yuen P.Yang, but differs in the plants sparsely spreading hyaline-pubscent in the upper part (vs. sparsely pubescent), leaf base triplinerved, lateral veins 3–5-pairs (vs. semi-basal triplinerved, lateral veins 2 or 3 pairs), stipules 4–6 mm long, purple-brown maculate (vs. 2–3 mm long, white-green, without minute spots), peduncles of staminate inflorescences 5–12 mm long, densely strigose (vs. 17–40 mm long, sparsely pubescent or glabrous), and achenes ca. 1 mm long, longitudinally 5-ribbed, tuberculate (vs. ca. 0.7 mm long, base lineolate and puncticulate).

**Figure 2.**
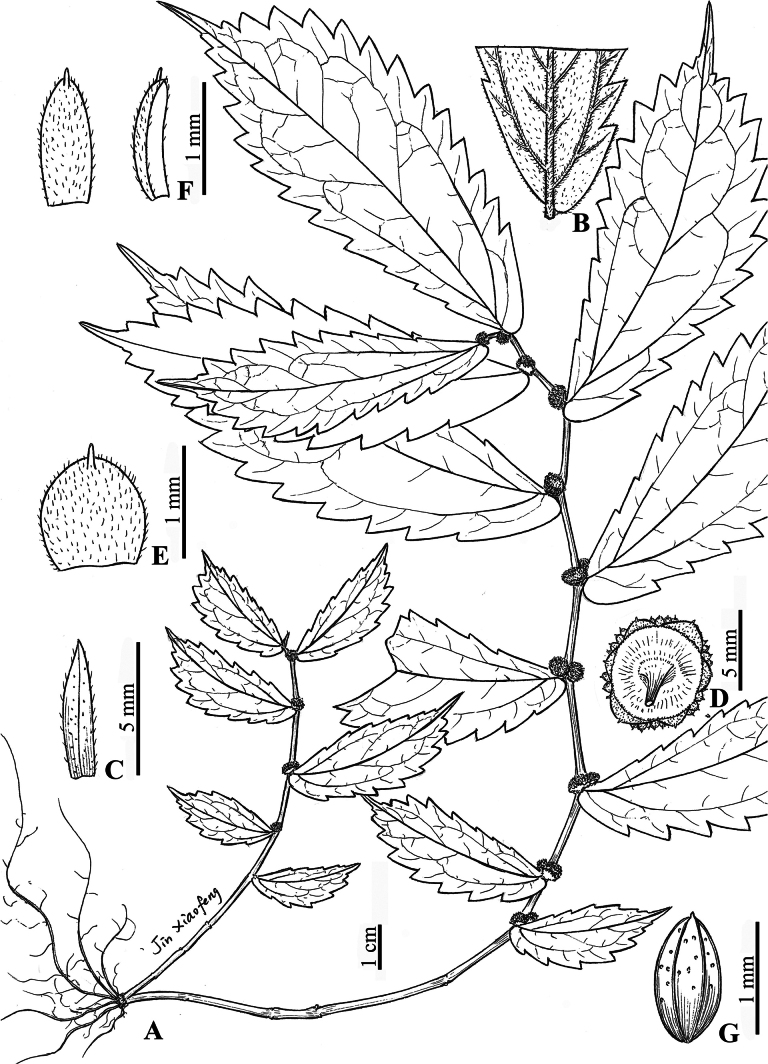
*Elatostema weizhianum* sp. nov. **A**. Habit; **B**. Lower part of leaf, showing abaxial indumentum and triplinerved base; **C**. Stipule; **D**. Receptacle, showing bracts; **E**. Outer bract; **F**. Bracteoles; **G**. Achene.

**Figure 3.**
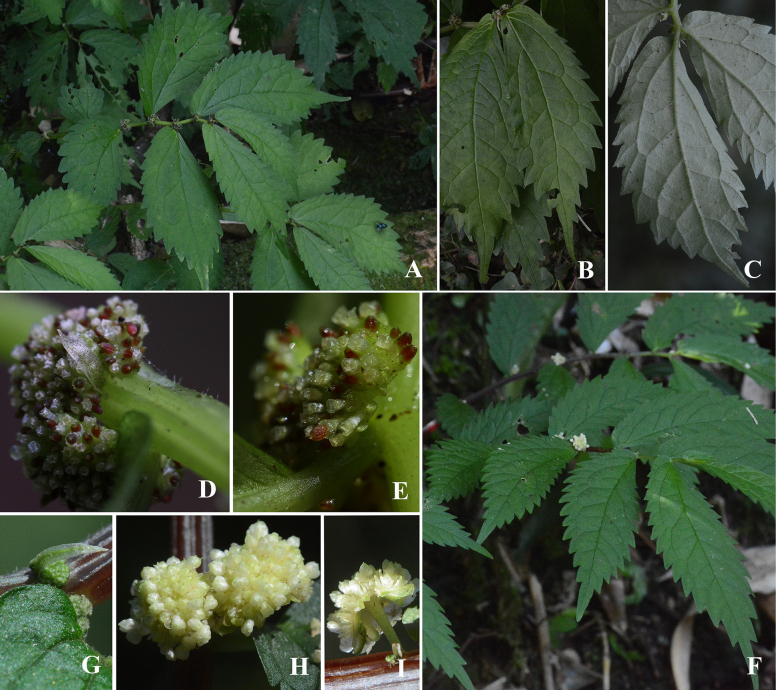
*Elatostema weizhianum* sp. nov. **A**. Habit of pistillate individual; **B**. Leaf, adaxial surface; **C**. Leaf, abaxial surface; **D**. Pistillate inflorescence and stipule; **E**. Pistillate inflorescence; **F**. Habit of staminate individual; **G**. Stipule; **H**. Staminate inflorescence; **I**. Staminate inflorescence, showing receptacle.

The new species is morphologically most similar to *E. grandidentatum* W.T.Wang in sharing triplinerved leaves with coarsely triangular-serrate margins and subsessile pistillate inflorescences, but differs in having stipules purple-brown maculate (vs. stipules white, without blotches), pistillate inflorescences borne in pairs axillary (vs. solitary), receptacle glabrous (vs. pubescent), bracts ca. 20, triangular, dorsally sparsely pubescent (vs. bracts 2, oblanceolate, densely pubescent), outer bracteoles ovate-orbicular, inner bracteoles oblong-navicular, apex corniculate, dorsally pubescent (vs. bracteoles monomorphic, lanceolate, glabrous), sepals inconspicuous, degenerative (vs. 3 distinct sepals), achenes 5-ribbed and tuberculate (vs. 13-ribbed, smooth).

##### Type.

China, Zhejiang: • Suichang, Mt. Jiulong, Huangjiping, along valley under forest, alt. 1450 m, 3 Aug 2019, *Y.L. Xu & al. 1572* (♀, holotype: ZM barcode ZMNH0067428; isotype: ZM barcode0067429).

##### Description.

Perennial herb, terrestrial, dioecious, with short tuberous root. Stems 10–40 cm tall, rarely branching, lower part glabrous, uppers sparsely spreading hyaline-pubescent, angulate (staminate individuals) or cylindrical (pistillate individuals). Leaves alternate, subsessile; blades membranous or thin-chartaceous, obliquely elliptic or obliquely obovate-elliptic, 2.5–17 cm long, 2–7 cm wide, apex obtuse, acute or cuspidate, base asymmetrical, broader-half round or auriculate, narrower-half cuneate, adaxially strigose, with cystoliths 0.3–0.4 mm long, abaxially hyaline-strigose, denser on veins, with cystoliths, margin triangular-dentate, ciliate, triplinerved, lateral veins 3–5 pairs. Stipules membranous, white, hyaline, linear-lanceolate, 4–6 mm long, 0.5–1 mm wide, purple-brown maculate; costa green, margin and adaxially pubescent, with cystoliths. Staminate inflorescences capitate, 1 or 2 axillary, pedunculate; peduncles 5–12 mm long, densely strigose, purple-brown maculate; receptacle sub-orbicular, ca. 4 mm in diam., glabrous, purple-brown maculate; bracts 6, with 2 larger ones opposite, broadly ovate-orbicular, ca. 4 mm long, ca. 5 mm wide, dorsally 3-carinate, lateral 2 pairs ca. 2 mm long, ca. 2 mm wide, dorsally 1-carinate; bracteoles linear-lanceolate, 2–2.5 mm long, 0.3–0.5 mm wide, glabrous. Staminate flowers pedicellate; pedicles ca. 2 mm long, glabrous; sepals 5, with 3 broader, ovate, ca. 1.5 mm long, ca. 0.8 mm wide, dorsally carinate, corniculate at apex, other 2 narrower, base connate; stamens 5. Pistillate inflorescences capitate, borne in pairs axillary, subsessile; receptacle sub-quadrate, 3–5 mm long, 3–5 mm wide, slightly 2–4-lobed, glabrous; bracts ca. 20, inconspicuous, short-corniculate; bracteoles membranous, outer layers ovate-orbicular, ca. 1 mm long, ca. 1 mm wide, apex round and corniculate, inner layers oblong-navicular, ca. 1 mm long, ca. 0.3 mm wide, apex corniculate, margin and dorsally pubescent; sepals inconspicuous, degenerative stamens 3 or 4, inflexed, stigma small. Achenes ellipsoid, purple-brown, ca. 1 mm long, with 5 longitudinal ribs, tuberculate.

##### Etymology.

The specific epithet “*weizhianum*” is given in honor of Prof. Zhi Wei, a Chinese taxonomist working at the Zhejiang Museum of Natural History, who studies vascular plants, especially the family Fabaceae.

##### Phenology.

Flowering and fruiting occurs from mid-June to early August.

##### Additional specimens examined.

China, Zhejiang: • Longquan, Mt. Fengyang, Dakeng, moist place along valley, alt. 1200 m, 17 Jun 2019, *Y.L. Xu & F.G. Zhang 1493* (♂, paratype: ZM).

##### Conservation status.

Endangered (EN). The new species is currently known from two localities, Mt. Jiulong in Suichang County and Mt. Fengyang in Longquan County. A total of 300 individuals in two populations grow in moist places along valleys, with an area of occupancy less than 200 km^2^. Referring to the IUCN Categories and Criteria, the species is endangered (EN, B2(a)) and requires conservation attention and protection (IUCN 2019).

##### Notes.

According to the taxonomic revision of *Elatostema* by Wang (2014), among 280 species documented in China, 60 species are known only from staminate inflorescences, and 82 species are known only from pistillate inflorescences. Both staminate (Mt. Fengyang) and pistillate (Mt. Jiulong) individuals of *E. weizhianum* were collected. The new species has capitate staminate inflorescences and a sub-orbicular, conspicuous receptacle, which indicates that the species should be placed in *E.* sect. *Elatostema* (Wang 2014). Wang divided *E.* sect. *Elatostema* into 26 series based on leaf venation, achene shape and surface sculpture, and pedicle length of staminate and pistillate inflorescences. The new species has achenes that are longitudinally 5-ribbed and tuberculate and should be assigned to *E.* ser. *Papillosa* W.T.Wang (Wang 2014).

## Supplementary Material

XML Treatment for Elatostema weizhianum

## Appendix 1

Taxa sampled and GenBank accession numbers (ITS, *psbA-trnH*, and *psbM-trnD*) of DNA sequences used in this study.

*Boehmeria densiflora*: —, KP858770, KP858509; *E. acuteserratum*: KP858860, KP858673, KP858600; *E. albopilosoides*: KP858885, KP858722, KP858577; *E. androstachyum*: KP858871, KP858711, KP858575; *E. asterocephalum*: KP858878, KP858712, KP858533; *E. australe*: KP858790, KP858732, KP858612; *E. balansae*: KP858847, KP858696, KP858559; *E. banahaense*: KP858815, KP858662, KP858592; *E. binatum*: KP858895, KP858726, KP858518; *E. brachyodontum*: KP858888, KP858708, KP858557; *E. calcareum*: KP858817, KP858650, KP858589; *E. cyrtandrifolium*: KP858842, KP858699, KP858531; *E. dissectum*: KP858832, KP858740, KP858607; *E. edule*: KP858818, KP858663, KP858590; *E. fengshanense*: KP858848, KP858694, KP858530; *E. ficoides*: KP858870, KP858703, KP858522; *E. fruticolosum*: KP858788, KP858733, KP858610; *E. garrettii*: KP858849, KP858698, KP858535; *E. goudotianum*: KP858798, KP858749, KP858629; *E. gracilipes*: KP858851, KP858675, KP858547; *E. grande*: KP858821, KP858655, KP858598; *E. grandidentatum*: KP858833, KP858737, KP858608; *E. grandifolium*: KP858823, KP858659, KP858595; *E. gyrocephalum*: KP858886, KP858727, KP858570; E. gyrocephalum var. pubicaule: KP858887, KP858728, KP858578; *E. hechiense*: KP858875, KP858729, KP858537; *E. hezhouense*: KP858881, KP858705, KP858538; *E. hirtellipedunculatum*: KP858863, KP858671, KP858568; *E. hookerianum*: KP858904, KP858735, KP858605; *E. huanjiangense*: KP858872, KP858730, KP858567; *E. hypoglaucum*: KP858891, KP858718, KP858516; *E. incisum*: KP858799, KP858750, KP858630; *E. insulare*: KP858839, KP858681, KP858554; *E. integrifolium*: KP858852, KP858676, KP858549; *E. involucratum*: KP858866, KP858672, KP858574; *E. japonicum*: KP858867, KP858715, KP858566; *E. kraemeri*: KP858824, KP858657, KP858596; *E. laetevirens*: KP858893, KP858683, KP858524; *E. laevissimum*: KP858861, KP858687, KP858562; *E. lineare*: KP858835, KP858713, KP858546; E. lineolatum var. major: KP858855, KP858689, KP858551; *E. lithoneurum*: KP858827, KP858653, KP858588; *E. lonchophyllum*: KP858793, KP858761, KP858512; *E. longistipulum*: KP858856, KP858679, KP858552; *E. lungzhouense*: KP858868, KP858716, KP858565; *E. lutescens*: KP858816, KP858651, KP858585; *E. macintyrei*: KP858850, KP858695, KP858560; *E. madagascariense*: KP858800, KP858752, KP858631; *E. malacotrichum*: KP858876, KP858710, KP858544; *E. microcephalanthum*: KP858873, KP858706, KP858556; *E. monandrum*: KP858905, KP858734, KP858606; *E. monticola*: KP858803, KP858690, KP858628; *E. morobense*: KP858820, KP858661, KP858593; *E. multicaule*: KP858884, KP858685, KP858520; *E. myrtillus*: KP858880, KP858704, KP858523; *E. nasutum*: KP858902, KP858741, KP858603; *E. oblongifolium*: KP858897, KP858723, KP858526; *E. obtusum*: KP858901, KP858739, KP858513; *E. oligophlebium*: KP858877, KP858721, KP858548; *E. orientale*: KP858802, KP858753, KP858627; *E. paivaeanum*: KP858801, KP858751, KP858625; *E. parvum*: KP858794, KP858744, KP858620; *E. penibukanense*: KP858854, KP858693, KP858542; *E. platyphyllum*: KP858858, KP858678, KP858540; *E. pusillum*: KP858906, KP858736, KP858602; *E. reticulatum*: KP858834, KP858714, KP858555; *E. rivulare*: KP858857, KP858697, KP858529; *E. samoense*: KP858826, KP858660, KP858594; *E. serra*: KP858837, KP858720, KP858534; *E. sinense*: KP858797, KP858747, KP858619; E. sinense var. longecornutum: KP858795, KP858745, KP858622; E. sinense var. xinningense: KP858796, KP858746, KP858621; *E. sinopurpureum*: KP858869, KP858686, KP858532; *E. strictum*: KP858825, KP858658, KP858597; *E. strigillosum*: KP858813, KP858667, KP858583; *E. subcoriaceum*: KP858894, KP858646, KP858539; *E. sublineare*: KP858882, KP858682, KP858514; *E. suzukii*: KP858864, KP858665, KP858525; *E. tenuicaudatum*: KP858862, KP858688, KP858528; *E. tenuinerve*: KP858896, KP858717, KP858572; *E. thalictroides*: KP858838, KP858680, KP858536; *E. tianeense*: KP858879, KP858647, KP858569;

*E. trilobulatum*: KP858900, KP858738, KP858515; E. variolaminosum var. latum: KP858791, KP858762, —; *E. villosum*: KP858812, KP858668, KP858582; *E. vitiense*: KP858789, KP858731, KP858611; *E. vittatum*: KP858792, KP858763, KP858609; *E. welwitschii*: KP858840, KP858691, KP858543; *E. xanthophyllum*: KP858890, KP858702, KP858571; *E. yachense*: KP858883, KP858684, KP858519; *E. yakushimense*: KP858892, KP858670, KP858517; *E. yaoshanense*: KP858903, KP858742, KP858604; *Lecanthus peduncularis*: KP858787, KP858645, KP858511; *Nanocnide japonica*: KP858907, KP858642, KP858505; *Pellionia grijsii*: —, —, KP858635; *Pellionia heterolobum*: KP858806, KP858756, KP858634; *Pellionia radicans*: KP858807, KP858743, KP858623; *Pellionia retrohispida*: KP858808, KP858760, KP858639; *Pellionia scabra*: —, —, KP858624; *Pellionia viride*: KP858805, KP858748, KP858633; *Poikilospermum acuminatum*: KP858908, KP858644, KP858510; *Procris archboldiana*: KP858785, KP858769, KP858614.

## Additional information

### Artificial Intelligence (AI) use

The authors accept full responsibility for the content of the manuscript, including the disclosure of any use of AI.

No AI tools were used in the preparation of this manuscript.

### Author ORCIDs

Y.-L. Xu https://orcid.org/0000-0002-4137-4397

Z.-H. Zheng https://orcid.org/0000-0001-5811-210X

Y.-F. Lu https://orcid.org/0000-0001-6095-3821

Z.-S. Tang https://orcid.org/0009-0005-0868-2944

X.-F. Jin https://orcid.org/0000-0002-8195-6738

### Data availability

All of the data that support the findings of this study are available in the main text.
