# Corrigenda: Xu YL, Zheng ZH, Lu YF, Tang ZS, Jin XF (2026) *Elatostema weizhianum*, a new species of Urticaceae from Zhejiang, East China. PhytoKeys 277: 65–75. https://doi.org/10.3897/phytokeys.277.200946

**DOI:** 10.3897/phytokeys.277.207971

**Published:** 2026-07-23

**Authors:** Yue-Liang Xu, Zi-Hong Zheng, Yi-Fei Lu, Zhan-Sheng Tang, Xiao-Feng Jin

**Affiliations:** 1 Zhejiang Museum of Natural History, Hangzhou, Zhejiang, 310012, China School of Forestry and Bio-technology, Zhejiang A&F University Hangzhou China; 2 Administration of Zhejiang Jiulongshan National Natural Reserve, Suichang, Zhejiang, 323300, China Zhejiang Museum of Natural History Hangzhou China; 3 School of Forestry and Bio-technology, Zhejiang A&F University, Hangzhou, Zhejiang, 311300, China Administration of Zhejiang Jiulongshan National Natural Reserve Suichang China

In the original publication of *Elatostema weizhianum* Y.L.Xu & X.F.Jin (Xu et al. 2026), the barcode numbers for the holotype and isotype were incorrectly recorded. The barcode number of the holotype should be corrected from ZMNH0067428 to ZMNH0067461, and that of the isotype from ZMNH0067429 to ZMNH0067462.

Herein, we provide the formal correction of the type below.

**Type**. China, Zhejiang: • Suichang, Mt. Jiulong, Huangjiping, along valley under forest, alt. 1450 m, 3 Aug 2019, *Y.L. Xu & al. 1572* ♀ (holotype: ZM barcode ZMNH0067461; isotype: ZM barcode ZMNH0067462).

## Additional information

### Artificial Intelligence (AI) use

The authors accept full responsibility for the content of the manuscript, including the disclosure of any use of AI.

No AI tools were used in the preparation of this manuscript.

### Author ORCIDs

Y.-L. Xu https://orcid.org/0000-0002-4137-4397

Z.-H. Zheng https://orcid.org/0000-0001-5811-210X

Y.-F. Lu https://orcid.org/0000-0001-6095-3821

Z.-S. Tang https://orcid.org/0009-0005-0868-2944

X.-F. Jin https://orcid.org/0000-0002-8195-6738

### Data availability

All of the data that support the findings of this study are available in the main text.
